# Lead toxicity induces autophagy to protect against cell death through mTORC1 pathway in cardiofibroblasts

**DOI:** 10.1042/BSR20140164

**Published:** 2015-03-31

**Authors:** Li Sui, Rui-Hong Zhang, Ping Zhang, Ke-Li Yun, Hong-Cai Zhang, Li Liu, Ming-Xu Hu

**Affiliations:** *Department of Emergency Medicine, The First Affiliated Hospital of Harbin Medical University, Harbin 150001, China; †Department of Cardiology, The First Affiliated Hospital of Harbin Medical University, Harbin 150001, China; ‡Department of Chinese Medicine, The First Affiliated Hospital of Harbin Medical University, Harbin 150001, China; §School of Food Science and Engineering, Harbin Institute of Technology, Harbin 150090, China; ║Academy of Traditional Chinese Medicine, Heilongjiang University of Chinese Medicine, Harbin 150040, China; ¶Department of Cardiology, the First Affiliated Hospital of Heilongjiang University of Chinese Medicine, Harbin 150040, China

**Keywords:** autophagy, cardiofibroblasts, endoplasmic reticulum (ER) stress, lead toxicity, mammalian target of rapamycin (mTOR) pathway, 3-MA, 3-methyladenine, DMEM, Dulbecco's Modified Essential Medium, ER, endoplasmic reticulum, LC3, light chain 3, mTOR, mammalian target of rapamycin, mTORC1, mammalian target of rapamycin complex 1, ROS, reactive oxygen species, S6K1, S6 kinase 1, UPR, unfolded protein response

## Abstract

Heavy metals, such as lead (Pb^2+^), are usually accumulated in human bodies and impair human's health. Lead is a metal with many recognized adverse health side effects and yet the molecular processes underlying lead toxicity are still poorly understood. In the present study, we proposed to investigate the effects of lead toxicity in cultured cardiofibroblasts. After lead treatment, cultured cardiofibroblasts showed severe endoplasmic reticulum (ER) stress. However, the lead-treated cardiofibroblasts were not dramatically apoptotic. Further, we found that these cells determined to undergo autophagy through inhibiting mammalian target of rapamycin complex 1 (mTORC1) pathway. Moreover, inhibition of autophagy by 3-methyladenine (3-MA) may dramatically enhance lead toxicity in cardiofibroblasts and cause cell death. Our data establish that lead toxicity induces cell stress in cardiofibroblasts and protective autophagy is activated by inhibition of mTORC1 pathway. These findings describe a mechanism by which lead toxicity may promote the autophagy of cardiofibroblasts cells, which protects cells from cell stress. Our findings provide evidence that autophagy may help cells to survive under ER stress conditions in cardiofibroblasts and may set up an effective therapeutic strategy for heavy metal toxicity.

## Abstract 2

Lead is a metal with many recognized adverse health side effects, and yet the molecular processes in cardiofibroblasts underlying lead toxicity are still poorly understood. Our current findings will help to understand the role of lead-mediated toxicity in cardiofibroblasts, indicating that autophagy serves a protective role in response to ER stress, which affords to set up an effective therapeutic strategy for the numerous diseases related to lead-toxicity.

## INTRODUCTION

Among heavy metals, lead (Pb^2+^) is regarded as a potent occupational toxin, but its toxicological manifestations are not well known. Lead toxicity is a particularly insidious hazard with the potential of causing irreversible health effects. It is known to interfere with a number of body functions and it is primarily affecting the central nervous, haematopoietic, hepatic and renal system producing serious disorders [1]. Lead contamination mainly occurs through absorption via the respiratory and gastrointestinal systems. Approximately 30%–40% of inhaled lead enters the bloodstream [2]. Once absorbed, 99% of lead is retained in the blood for approximately 30–35 days and, over the following 4–6 weeks, it is dispersed and accumulated in other tissues [3]. However, the molecular mechanisms of lead toxicity are still not clearly defined. The effects of lead on calcium fluxes and calcium-regulated events have been suggested as major mechanisms of lead neurotoxicity [4]. Another potential mechanism of lead toxicity is the ability of lead to induce oxidative stress. The deleterious effects of lead exposures can involve both the generation of reactive oxygen species (ROS) and a direct depletion of the antioxidant reserves [3]. ROS production and the generation of other potentially genotoxic compounds are possible mechanisms of the carcinogenicity of lead. Significant effects have been found on various fundamental cellular processes, like protein folding and maturation, apoptosis, ionic transportation and enzyme regulation [5], leading to cell stress and finally cell death.

Earlier reports have indicated that heavy metals may cause endoplasmic reticulum (ER) stress to cultured vascular endothelial cells [6], neuroblastoma (SH-SY5Y) cells [7] and porcine renal epithelial cell line (LLC-PK1) renal epithelial cells [8]. ER is an organelle that has an essential role in multiple cellular processes, such as the folding of secretory and membrane proteins, calcium homoeostasis and lipid biosynthesis. When ER transmembrane sensors detect the accumulation of unfolded proteins, the unfolded protein response (UPR) is initiated to cope with the resulting ER stress [9]. When the ER stress is extensive or sustained and the function of ER cannot be restored, it also initiates apoptotic signalling [10].

Under stress conditions, cell performs several adaptive alternations to protect against cell death, such as autophagy. Autophagy is a highly conserved cellular process in which cytoplasmic materials, including organelles, are sequestered into double-membrane vesicles called autophagosomes and delivered to lysosomes for degradation or recycling [11]. Autophagy is a tightly regulated pathway, which can be stimulated by multiple forms of cellular stress, including nutrient or growth factor deprivation, hypoxia, ROS, DNA damage, protein aggregates, damaged organelles or intracellular pathogens [12]. The stimulation of autophagy by these stimuli involves diverse signals that have overlapping functions in autophagy and the control of other cellular stress responses. The regulation of autophagy is a very complex process which includes many signalling pathways, such as the phosphatidylinositol 3-kinase-I (PI3K) and mammalian target of rapamycin (mTOR) pathway and mitogen-activated protein kinase (MAPK) pathways [13,14]. It was reported that autophagy serves as a protective mechanism against cell death during nutrient deprivation and cells undergo apoptotic cell death when autophagy is inhibited [15,16]. However, autophagy has also been observed as a cell death, no matter caspase is activated or not. Though the molecular mechanism of apoptosis is well documented, autophagy remains not well-understood, especially for the intrinsic and extrinsic mediators of autophagy.

Although heavy metals have been reported to cause cell stress and even cell death in some types of cells, the detailed toxicity and molecular mechanisms of heavy metals on mammalian cells are still largely unknown. Moreover, it also need to determine the cell responses to heavy metals, which may help to clarify the signalling molecular pathways of heavy metals on mammalian cells and to design targeted drugs. In the present study, we focused on lead (Pb^2+^), a significant heavy metal with cell toxicity and proposed to determine, (i) whether lead may induce the cell stress and cell death in cardiofibroblast cells, (ii) how cells response to lead toxicity to survive under toxic conditions. Our results show that lead treatment may induce robust UPR and ER stress in cardiofibroblast cells and lead-mediated autophagy helps cells to survive from the toxicity. Moreover, lead treatment inhibits mTOR complex 1 (mTORC1) pathway, which contributes to the autophagy activation. Inhibited autophagy enhances lead toxicity and causes robust cell death. Our work will help to understand the role of lead-mediated toxicity in mammalian cells, indicating that autophagy serves a protective role in response to ER stress, which affords to open new possibilities for the treatment of the numerous diseases related to lead-toxicity.

## MATERIALS AND METHODS

### Reagents and antibodies

The plumbous acetate [Pb(Ac)_2_] for Pb^2+^ supply was purchased from Sigma–Aldrich. Dulbecco's Modified Essential Medium (DMEM) and FBS were purchased from GIBCO Invitrogen. The Lyso-Tracker Red probes for acidic lysosome staining were from Beyotime. Anti-GRP78 (glucose-regulated protein 78), anti-GRP94, phosphorylates eukaryotic initiation factor 2 (anti-peIF2a), anti-cleaved caspase-3, anti-light chain 3 (anti-LC3) and anti-pS6 were purchased from Cell Signaling Technology. Anti-GAPDH (glyceraldehyde-3-phosphate dehydrogenase), BCL2-associated X protein (anti-Bax), B-cell lymphoma 2 (anti-BcL2) and anti-p70S6K (S6 kinase 1) antibodies were from Millipore. The p62 antibody was from Santa Cruz. Other reagents were of the highest purity available.

### Cell culture

The cardiac fibroblasts were isolated from about 4-week-old wild-type mice as previously described [17]. The experimental mice (C57BL/6) were obtained from the Experimental Animal Center of Harbin Medical University. The protocols of feeding were formed in accordance with the Guidelines of Institute of Harbin Medical University Animals Research Committee. Briefly, the mice were killed and the hearts were dissected free from extra-cardiac tissue and atria. Hearts were rinsed in saline solution, minced and digested with collagenase type 2 (Worthington Biochemical). Isolated fibroblasts were then purified by selective attachment to tissue culture plastic. Cells were cultured in DMEM containing 15% FBS, anti-mycotics and antibiotics in 5% CO_2_ at 37°C.

### Pharmacological manipulations

For Pb^2+^ treatment, we applied Pb^2+^ to these cells at the final concentration from 0, 1, 10 and 100 nM for 12 h. Cells with no additive were used as internal controls. Then the cells were harvested for subsequent mRNA analysis. To further study the role of Pb^2+^ on ER stress and autophagy in cells, treatment of Pb^2+^ at 100 nM from 0, 6, 12 and 24 h were applied to cells for mRNA and protein analysis.

### Lyso-Tracker Red staining

To detect the autophagy status in Pb^2+^-treated cardiac fibroblast cells, we applied Lyso-Tracker Red staining to examine the acidic lysosomes. For the preparation of Lyso-Tracker Red staining, cells were plated with 1.0×10^5^ cells/ml in six-well plates. After Pb^2+^ treated at the final concentration of 100 nM for different times, living cells were directly stained with Lyso-Tracker Red probes from Beyotime following as instructions.

### Assay of real-time PCR

Total RNA was isolated from cardiac fibroblasts using Trizol reagent (Invitrogen). RNA was subjected to reverse transcription with reverse transcriptase as manufacturer's instructions (Fermentas). Quantitative real-time PCR was performed using the Bio-Rad iQ5 system and the relative levels of gene expression were normalized to internal control as β-actin. Primer sequences for SYBR Green probes of target genes were as following, Grp78: CGCTTCGAATCGGCGGTACCCAG and TCCTTCTTGTCCTCCT CCTAAGCTTCGCG; Grp94: CAGTTTTGGATCTTGCTGTGG and CAGCTGTAGA TTCCTTTGC; Xbp1-s (X-box-binding protein 1-s): GAACCAGGAGTTAAGAACAC and AGGCAACAGTGTC AGAGTCC; β-actin: AGAGGGAAATCGTGCGTGAC and CAATAGTGATGACC TGGCCGT.

### Assay of Western blots

Cell samples were extracted in lysis buffer containing 1% Triton X-100, 75 mM NaCl, 5 mM Tris (pH 7.4), 0.5 mM orthovanadate, 0.5 mM EDTA, 0.5 mM EGTA, 0.25% NP-40 and protease inhibitors. Total protein concentrations in each sample were determined using the BCA (Pierce Biotechnology). Equal amounts of lysate (25–50 μg) were separated on a SDS/PAGE (10% gel; BioRad Laboratories). Proteins were electrophoretically transferred to nitrocellulose membranes, which were then stained with Ponceau Red to verify even transfer. Membranes were blocked in 5% powdered milk resuspended in TBS and then incubated with appropriate antibodies. Secondary antibodies were goat anti-rabbit or anti-mouse coupled to HRP (horseradish peroxidase; Pierce Biotechnology). Blots were developed using SuperSignal reagents (Pierce Biotechnology), exposed to X-ray film and immunoreactive bands were quantified using the ImageJ software.

### Statistical methodology

All statistical analysis was performed by Image software. Quantitative data were showed in x^−^ ± s using *t* tests for comparisons. The value 0.05 (*), 0.01 (**) and 0.001 (***) was assumed as the level of significance for the statistic tests carried out.

## RESULTS

### Effects of lead toxicity on the expression of UPR genes in cardiofibroblasts

**Figure 1:**
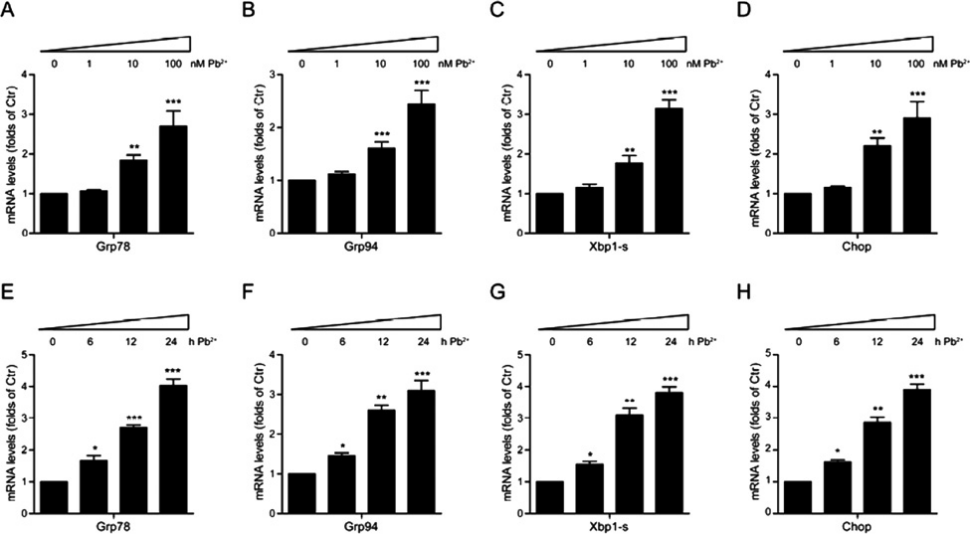
Lead (Pb^2+^) induces UPR gene transcriptions in cardiofibroblasts (**A**–**D**) Real-time PCR results showing the increasing of UPR gene transcriptions, *Grp78* (**A**), *Grp94* (**B**), *Xbp1-s* (**C**) and *Chop* (**D**) in cardiofibroblasts after Pb^2+^ treatment with different dosages (0–100 nM) for 24 h. Results are averages of three independent experiments. Data represent mean ± S.E.M. **P<0.01, ***P<0.001. (**E**–**H**) Real-time PCR results showing the increasing of UPR gene transcriptions, *Grp78* (**E**), *Grp94* (**F**), *Xbp1-s* (**G**) and *Chop* (**H**) in cardiofibroblasts after Pb^2+^ treatment (100 nM) in different time points (0–24 h). Results are averages of three independent experiments. Data represent mean ± S.E.M. *P<0.05, **P<0.01, ***P<0.001.

To investigate the lead toxicity on cultured cardiofibroblasts, we firstly examined that whether lead would induce ER stress. The cardiofibroblasts were exposed to Pb^2+^ of different concentrations (0∼100 nM) by different treated times (0∼24 h). As shown in Figure 1, results of real-time PCR suggested that the expression of *Grp78, Grp94, Xbp1-s* and CCAAT/enhancer-binding protein homology protein (*Chop*) mRNA increased significantly in response to lead concentrations of 1, 10 and 100 nM in a dose-dependent manner (Figures 1A–1D). When cells were incubated with 100 nM Pb^2+^ for 0, 6, 12 and 24 h, the *Grp78, Grp94, Xbp1-s and Chop* levels increased in a time-dependent manner (Figures 1E–1H). Although these UPR genes show different changes under the Pb^2+^ exposure, the expression of UPR genes in cardiofibroblasts were all significantly enhanced, suggesting that UPR may reflect lead toxicity in cardiofibroblasts. Thus, different expression of these UPR genes are highly correlated to different Pb^2+^ concentrations and treatment times, in a time- and dose-dependent manner. Collectively, these findings suggested that lead significantly activates ER stress, which may potentially impair regular cell metabolism and survival.

### Effects of lead toxicity on the expression of UPR proteins in cardiofibroblasts

**Figure 2:**
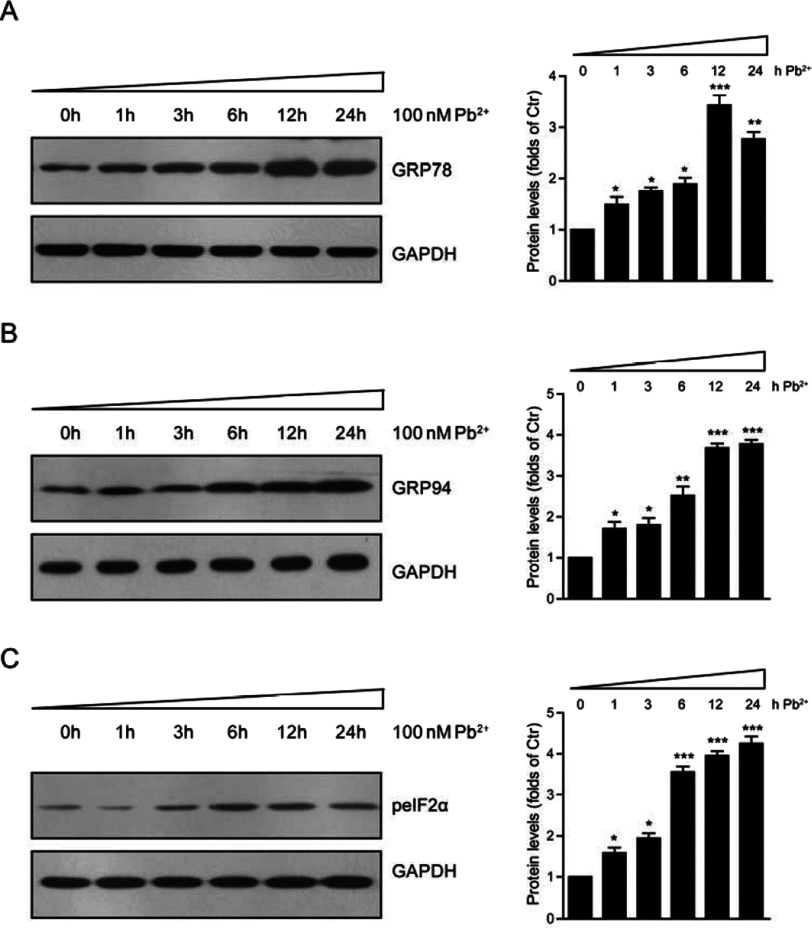
Lead (Pb^2+^) induces UPR protein levels in cardiofibroblasts Western blots and histograms showing that UPR protein levels, GRP78 (**A**), GRP94 (**B**) and peIF2α (**C**), increased in cardiofibroblasts after Pb^2+^ treatment (100 nM) in different time points (0–24 h). Results are averages of three independent experiments. Data represent mean ± S.E.M. *P<0.05, **P<0.01, ***P<0.001.

To confirm the lead toxicity in cardiofibroblasts, we next examined the UPR protein levels by lead treatment, such as GRP78, GRP94 and peIF2α. As shown in Figure 2, the expression of GRP78 protein increased significantly up to 12 h with maximum increases of 3.44-folds compared with the control (Figure 2A). The expression of GRP94 protein increased progressively up to 24 h, the highest value increasing by 3.78-folds compared with the control (Figure 2B). The levels of peIF2α protein showed maximum increases of 4.26-folds at 24 h (Figure 2C). Thus, our results showed that Pb^2+^ in the medium really influenced the expressions of GRP78, GRP94 and peIF2α in a time-dependent manner, which may be the results of commonly ER response to lead toxicity. Induction of GRP78, GRP94 and peIF2α expression has previously been reported in cells exposed to heavy metals toxicity [6,8]. Compared with previous studies, our findings showed that lead toxicity may robustly induce ER stress in cardiofibroblasts [18].

### Lead toxicity does not induce dramatic cell death in cardiofibroblasts

**Figure 3:**
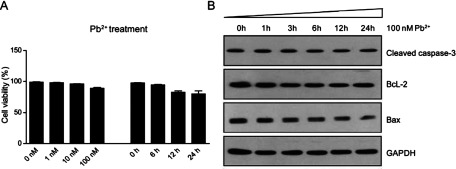
Lead (Pb^2+^) does not induce dramatic cell death in cardiofibroblasts (**A**) Results of MTT assays showing that cell viability is not reduced dramatically by either different dosages (0–100 nM) of Pb^2+^ for 24 h (left) or different time points (0–24 h) of Pb^2+^ by 100 nM (right). (**B**) Western blots showing that apoptosis-related proteins, cleaved caspase-3, BcL-2 and Bax, are not altered by Pb^2+^ treatment (100 nM) in different time points (0–24 h) in cardiofibroblasts.

Severe ER stress impairs normal cell structure and functions, which may lead to cell death. For we have identified that lead toxicity could induce ER stress in cardiofibroblasts, it is of high possibility that lead toxicity would increase cell death in these cells. To further determine the cell death by lead treatment, we applied MTT assay to detect cell viability of cardiofibroblasts by lead treatment. Unexpectedly, the results showed that there was no dramatic cell death in cardiofibroblasts by lead treatment (Figure 3A). Moreover, the biochemical results also showed that lead treatment could not alter protein levels of cleaved caspase-3, Bax and BcL-2, which were all the indicators of cell apoptosis (Figure 3B). Although lead treatment induces severe ER stress in cardiofibroblasts, the cell viability seems not be affected by lead toxicity. Thus, cardiofibroblasts may perform some adaptive changes to survive by lead treatment.

### Lead toxicity induces autophagy in cardiofibroblasts

**Figure 4:**
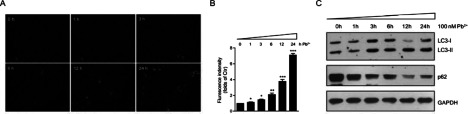
Lead (Pb^2+^) induces autophagy in cardiofibroblasts (**A**) Acidic lysosome staining by Lyso-Tracker Red probes showing the increasing of autophagy by Pb^2+^ treatment (100 nM from 0 to 24 h) in living cardiofibroblasts. (**B**) Histograms showing the quantifications of lysosome staining (**A**) fluorescence intensity. Results are averages of three independent experiments. Data represent mean ± S.E.M. *P<0.05, **P<0.01, ***P<0.001. (**C**) Western blots showing the protein levels of LC3-I/II and p62 are altered by Pb^2+^ treatment (100 nM from 0 to 24 h) in cardiofibroblasts.

It is a surprise that cardiofibroblasts were not dramatically apoptotic by lead treatment, although they suffered from serious ER stress. Therefore, lead treatment may also induce protective adaptations in cardiofibroblasts. To study how cultured cardiofibroblasts resist ER stress for cell survival, we focused on an important cellular protective mechanism against apoptosis, autophagy. To examine whether the autophagy was increased in mouse cardiofibroblasts exposed to lead toxicity, the living lysosome staining was assayed using these cardiofibroblasts. The Lyso-Tracker Red probes may afford highly selective staining of acidic lysosomes, which are recognized as markers of autophagy. Our results show that the staining intensity of Red-Fluorescence gradually increases in cultured cardiofibroblasts with the increased times up to 24 h, the highest value being about 7.01-folds of control (Figures 4A and 4B). To confirm the increased autophagy in Pb^2+^-treated cardiofibroblasts, we carried out biochemical assays to examine the LC3 expression levels in cardiofibroblasts exposed to Pb^2+^. The LC3-II:LC3-I ratio, as determined by Western blotting, is a commonly used measure of autophagosome levels [18]. In the present study, our results suggested that LC3-I form gradually converted into LC3-II form, which supports the notion that Pb^2+^ treatment mediates autophagy in cardiofibroblasts (Figure 4C). Moreover, we examined another indicator of autophagy, p62. It has been accepted that p62 protein reduction is a key event for autophagy activation. By Western blots, we found that p62 protein level was reduced after Pb^2+^ treatment (Figure 4C). These result combined with lysosome staining supports that Pb^2+^ indeed enhances mediates autophagy in cardiofibroblasts [18].

### Lead toxicity mediates autophagy by inhibiting mTORC1 pathway in cardiofibroblasts

**Figure 5:**
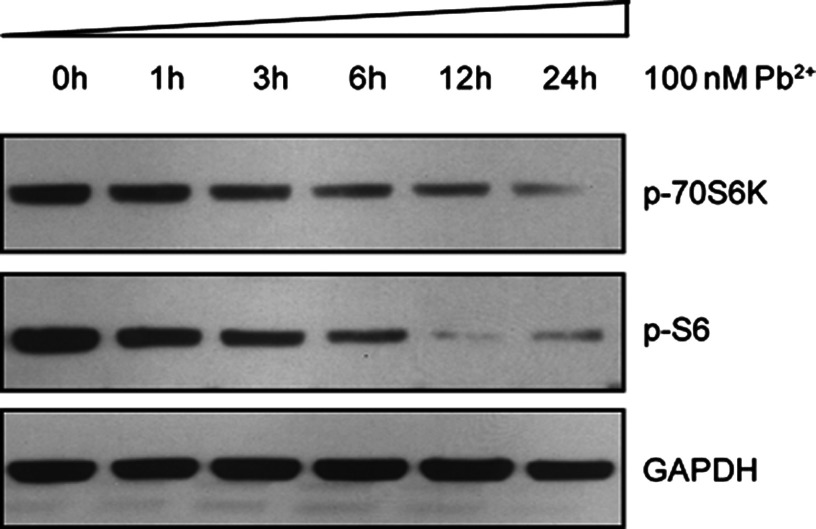
Lead (Pb^2+^) inhibits mTORC1 activity in cardiofibroblasts Western blots showing that the protein levels mTORC1 downstream markers p-70S6K and p-S6 were decreased in cardiofibroblasts by Pb^2+^ treatment (100 nM from 0 to 24 h) in cardiofibroblasts.

To reveal the mechanism of how lead toxicity mediates autophagy in cardiofibroblasts, we examined the activity of mTORC1 signalling after lead treatment. mTORC1 is widely accepted as a master regulator of autophagy *in vitro* and *in vivo*. Inhibition mTORC1 activity by rapamycin could effectively induce autophagy [20]. In mammalian cells, mTORC1 phosphorylates proteins involved in protein synthesis and cell growth, including p70S6K1, which phosphorylates the ribosomal protein S6. By Western blots, we found that p70S6K and pS6, which were markers of mTORC1 activity, were gradually reduced after Pb^2+^ treatment (100 nM from 0 to 24 h; Figure 5). These results strongly showed that Pb^2+^ inhibits mTORC1 activity, which may contribute to the increasing of autophagy in cardiofibroblasts.

### Inhibition of autophagy promotes lead-induced cell death in cardiofibroblasts

**Figure 6:**
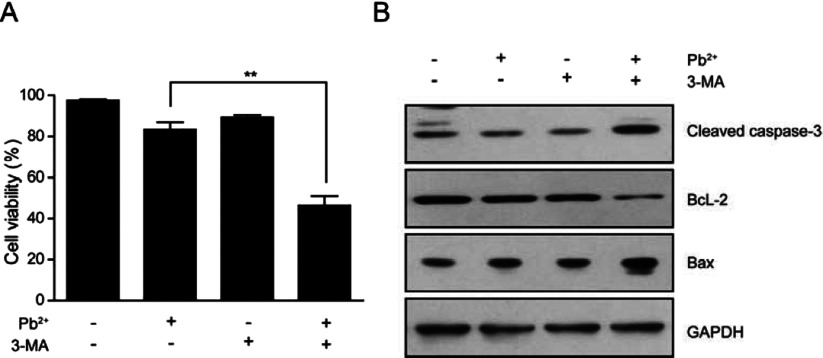
Inhibition of autophagy promotes lead-induced cell death in cardiofibroblasts (A) Results of MTT assays showing that cell viability is reduced dramatically by Pb^2+^ and 3-MA co-treatment. Results are averages of three independent experiments. Data represent mean ± S.E.M. **P<0.01. (**B**) Western blots showing that apoptosis-related proteins, cleaved caspase-3, BcL-2 and Bax, are altered by Pb^2+^ and 3-MA co-treatment in cardiofibroblasts.

We have identified that lead treatment activates autophagy by inhibiting mTORC1 activity, which may play a key role in the resistance of cell death. To further confirm the role of autophagy in the resistance of lead toxicity, we designed to inhibit autophagy and investigate whether this would enhance lead toxicity on cell death in cardiofibroblasts. We applied autophagy inhibitor 3-MA to lead treated cardiofibroblasts and detected the cell viability. Although cell viability was not affected by lead treatment alone, the 3-MA (3-methyladenine) could reduce cell viability dramatically in the presence of lead (Figure 6A). Moreover, we examined the indicators of cell apoptosis and found that all the protein levels (cleaved caspase-3, Bax and BcL-2) were dramatically altered by lead and 3-MA co-treatment (Figure 6B). Collectively, these results clearly showed that inhibition of autophagy may enhance lead toxicity in cardiofibroblasts and cause cell death.

## DISCUSSION

**Figure 7:**
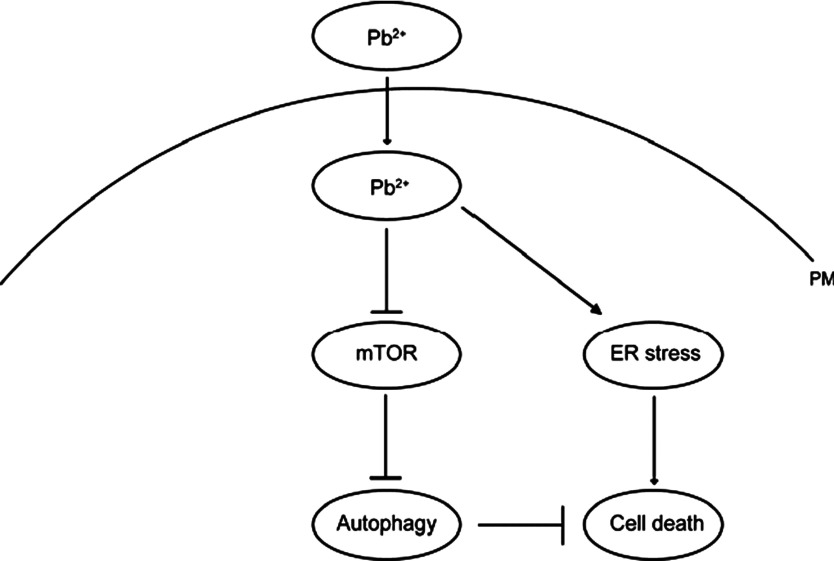
Models Schematic representation highlighting the molecular link between Pb^2+^, ER stress, autophagy and cell death in cardiofibroblasts. Pb^2+^ induces ER stress in time- and dose-dependent manners. In the meantime, Pb^2+^ activates autophagy by inhibiting mTORC1 activity, which would contribute to the protection of cardiofibroblasts from cell death. Abbreviation: PM, plasma membrane.

Evidence is emerging that the heavy metals, such as lead, has toxicity to mammalian cells. In the present study, we demonstrate that lead-toxicity dramatically induces UPR and ER stress in cardiofibroblasts. To protect cells for survival, lead-mediated autophagy is activated by mTORC1 pathway inhibition, which releases cell stress and promotes cell survival. Our work uncovers novel network on the inter-relationship between lead toxicity, ER stress, autophagy and cell death, which are summarized in Figure 7.

ER is the site of folding of membrane and secreted proteins in the cell. Physiological or pathological processes that disturb protein folding in the ER cause ER stress and activate a set of signalling pathways termed the UPR [21]. The UPR can promote cellular repair and sustained survival by reducing the load of unfolded proteins through up-regulation of chaperones and global attenuation of protein synthesis. Our present studies have revealed that lead, a toxic metal, may dramatically induce ER stress, which may impair ER structure and functions. The targeted lead toxicity in the ER may disrupt normal protein synthesis and many other cellular events. This may partly help to explain the cell toxicity of heavy metals in mammalian cells.

Notably, when the ER stresses are overloaded, other cell protective mechanisms are started, such as autophagy [22]. The biological link between ER stress and autophagy was reported previously, but the mediators of these two events were not well-understood [23]. In the present study, we firstly exhibit the network between heavy metal toxicity, ER stress and autophagy. Lead, a significant environmental toxin, has been showed to mediate UPR and autophagy in cardiofibroblasts. Fortunately, these cells survive by lead-mediated autophagy, confirming that autophagy effectively protects cells from stress or toxicity conditions. The future research will deeply clarify the relationship between lead toxicity, ER stress and autophagy, in that how the intracellular lead toxicity mediates ER stress and autophagy, paving the way for pharmacological exploitation of signalling pathways involved.

The regulation of translation and autophagy is mediated by mTORC1 and our work for the first time uncloses that lead toxicity could inactivate mTORC1 pathway in a time-dependent manner, which contributes to the autophagy activation. Besides cell autophagy, mTORC1 controls cell size [24], proliferation [25] and metabolism [26]. On the other hand, as for mTORC1 activity is critical for new protein synthesis, the reduced mTORC1 activity may decrease new protein synthesis, which may partly attenuate ER stress on protein folding and processing reactions. Given that lead toxicity could inhibit mTORC1 pathway, we would like to investigate whether the cell development, proliferation and energy production would be disrupted by lead treatment.

In summary, our current findings showed that autophagy is mediated by lead toxicity, which protect cardiofibroblasts from ER stress. To our knowledge, this is the first report of lead toxicity correlated with ER stress and autophagy in cardiofibroblasts. Further study will be needed to gain additional details about the regulatory association of how intracellular Pb^2+^ induces cell stress, autophagy and cell death and set stages to the clinical treatment of related diseases.
